# Hospital effluent: A reservoir for carbapenemase-producing *Enterobacterales*?

**DOI:** 10.1016/j.scitotenv.2019.03.428

**Published:** 2019-07-01

**Authors:** Niamh Cahill, Louise O'Connor, Bláthnaid Mahon, Áine Varley, Elaine McGrath, Phelim Ryan, Martin Cormican, Carina Brehony, Keith A. Jolley, Martin C. Maiden, Sylvain Brisse, Dearbháile Morris

**Affiliations:** a Antimicrobial Resistance and Microbial Ecology Group, School of Medicine, National University of Ireland, Galway, Ireland; b Centre for Health from Environment, Ryan Institute, National University of Ireland, Galway, Ireland; c Carbapenemase-Producing *Enterobacterales* Reference Laboratory, Department of Medical Microbiology, University Hospital Galway, Galway, Ireland; d Department of Zoology, University of Oxford, Oxford, United Kingdom; e Biodiversity and Epidemiology of Bacterial Pathogens, Institut Pasteur, Paris, France

**Keywords:** Antimicrobial resistant bacteria, Carbapenem resistance, Hospital wastewater, Municipal wastewater, Carbapenemase-encoding genes

## Abstract

Antimicrobial resistance is a major public health concern. Carbapenemase-producing *Enterobacterales* (CPE) represent a significant health threat as some strains are resistant to almost all available antibiotics. The aim of this research was to examine hospital effluent and municipal wastewater in an urban area in Ireland for CPE. Samples of hospital effluent (*n* = 5), municipal wastewater before (n = 5) and after (*n* = 4) the hospital effluent stream joined the municipal wastewater stream were collected over a nine-week period (May–June 2017). All samples were examined for CPE by direct plating onto Brilliance CRE agar. Isolates were selected for susceptibility testing to 15 antimicrobial agents in accordance with EUCAST criteria. Where relevant, isolates were tested for carbapenemase-encoding genes by real-time PCR. CPE were detected in five samples of hospital effluent, one sample of pre-hospital wastewater and three samples of post-hospital wastewater. Our findings suggest hospital effluent is a major contributor to CPE in municipal wastewater. Monitoring of hospital effluent for CPE could have important applications in detection and risk management of unrecognised dissemination of CPE in both the healthcare setting and the environment.

## Highlights

• The impact of untreated hospital effluent on municipal sewage was investigated.
• Higher prevalence of CPE detected in hospital effluent versus municipal sewage.
• blaOXA-48, blaKPC, blaVIM and blaIMP present in hospital and post-hospital sewage
• blaNDM present in municipal sewage
• Releasing untreated hospital sewage may fuel CPE dissemination in the environment.

## Graphical abstract

**Figure:**
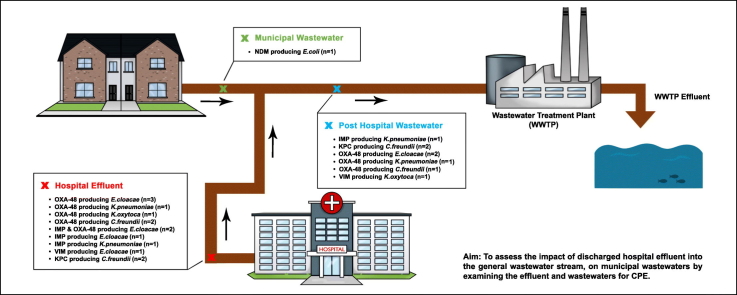

## 1 Introduction

Antimicrobial resistance is a major public health concern worldwide. Although it is by no means a new phenomenon, it is one which is continuously escalating (CDC, 2013; WHO, 2018). In May 2015, the World Health Assembly developed a global action plan to tackle the problem of antimicrobial resistance (AMR) (WHO, 2015). In June 2017, the European Commission published the EU One Health Action Plan against AMR which builds on the EU Action plan against AMR previously launched in 2011. The new action plan against AMR advocates a one health approach, and highlights the importance of the environment, human health and animal health interconnection (European Commission, 2017). In October 2017, the Irish government published Ireland's National Action Plan on Antimicrobial Resistance 2017–2020 (Department of Health, 2017), and CPE was declared a public health emergency.

Carbapenemase-producing *Enterobacterales* (CPE) have been identified in recent years as one of the major threats to human health. CPE are included in the WHO's critically important priority list of pathogens for which new antibiotics are required (CDC, 2013; Tacconelli et al., 2018; WHO, 2017). CPE are of particular concern, as in some cases of infection with CPE there are very limited treatment options available, e.g. colistin, tigecycline and fosfomycin. Unfortunately reports of resistance to last resort antibiotics are emerging. This includes the recently reported findings of plasmid-encoded colistin resistance (*mcr*-*1*) initially in pigs in China, and subsequently in humans and animals worldwide (Caniaux et al., 2017). The detection of other MCR variants have also been reported, including the novel variant *mcr*-*8*, which has been found present in both human and animal samples also (Wang et al., 2018).

Carbapenemases belong to all four Ambler molecular classes, and the most commonly reported enzymes include *Klebsiella pneumoniae* carbapenemases (KPC), verona-integron-encoded metallo-β-lactamases (VIM), imipenem-resistant *Pseudomonas* (IMP), New Delhi metallo β-lactamase (NDM) and the Class D carbapenem-hydrolyzing oxacillinase (OXA-48) (Nordmann et al., 2011). The most commonly reported carbapenemases in Ireland are OXA-48, KPC and NDM (NCPERLS, 2018). In Ireland, at present, the number of blood stream infections caused by CPE is relatively low. Fifteen cases were reported in 2018, however, this is an increase from nine in 2017 (Unpublished data; NCPERLS). The number of people identified as colonised with CPE has increased from 282 in 2016, to 433 in 2017 (NCPERLS, 2017; NCPERLS, 2018), to 537 in 2018 (Unpublished data; NCPERLS).

Although healthcare facilities are the primary reported reservoirs of antimicrobial resistant bacteria (ARB) (Galler et al., 2014); other reservoirs have also been identified, such as animals and the environment which may contribute to the worldwide dissemination and persistence of ARB including pandemic clonal groups such as *Escherichia coli* ST131 and *Klebsiella pneumoniae* ST258 (Caltagirone et al., 2017; Colomer-Lluch et al., 2013; Ewers et al., 2010; Woodford et al., 2014). The detection of ARB in wastewater has been reported previously, highlighting its potential role as a transmission route for ARB to the environment (Galler et al., 2014; Hocquet et al., 2016; White et al., 2016). A high abundance of ARB and antimicrobial resistant genes (ARG), including clinically significant extended-spectrum beta-lactamase (ESBL) and carbapenemase-encoding genes, have been detected in hospital effluent previously (Haller et al., 2018; Lamba et al., 2017; Nasri et al., 2017). As hospital effluent is generally released untreated into the main wastewater stream and subsequently into the environment this is a cause for concern. Overall, very little research comparing the levels of ARB and ARG in hospital effluent to municipal wastewater containing no hospital effluent has been carried out. Some studies which have assessed this have reported lower levels of ARB and ARG in municipal wastewater in comparison to hospital effluent (Islam et al., 2017; Lamba et al., 2017). In studies conducted by Azuma et al. (2019) and Proia et al. (2018), hospital effluent, wastewater treatment plant (WWTP) influent and effluent, as well as rivers receiving WWTP effluent were assessed for the presence of ARB and ARG, with both reporting the detection of ARB and ARG in all locations sampled. Findings from these studies investigating the impact of HE and WWTP effluents on the receiving aquatic environments suggest that dissemination of ARB and ARG is potentially being fuelled by the release of untreated or inadequately treated hospital effluent into the environment (Azuma et al., 2019; Proia et al., 2018). Khan et al. (2018) reported the detection of carbapenemase-producing *Klebsiella oxytoca* in WWTP influent containing hospital effluent, as well as in the WWTP effluent receiving river in Sweden. VIM-producing *K. oxytoca* detected in the WWTP effluent receiving river was found to genotypically match VIM-producing *K. oxytoca* isolates obtained from human tissue and urine specimens in a local Swedish hospital (Khan et al., 2018). Such findings highlight the dissemination of these clinically significant ARB from hospitals into the environment (Khan et al., 2018). The presence, persistence and dissemination of clinically significant ARB and ARG in the environment is a cause of concern for public health as aquatic environments are commonly used for recreational, irrigation and drinking water purposes.

The aim of this study was to examine municipal wastewaters in an urban area in Ireland for CPE and the impact discharge of hospital effluent directly to the wastewater stream may have on this.

## 2 Materials and methods

### 2.1 Sample collection, isolation and characterisation of CPE

**Fig. 1:**
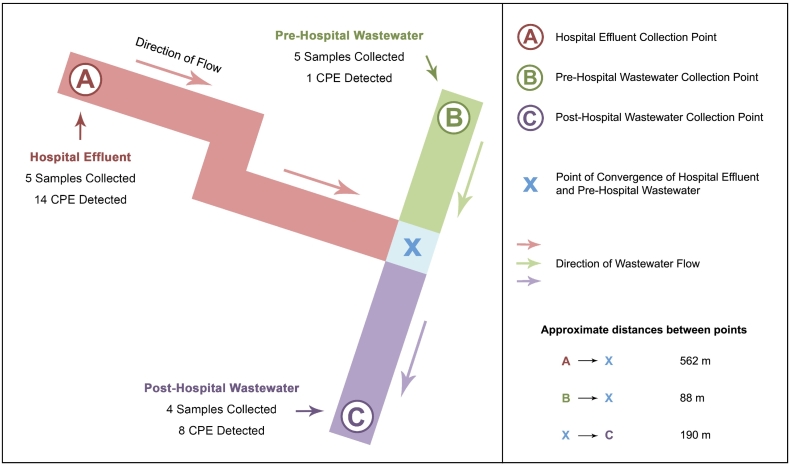
Schematic map illustrating wastewater collection points.

Samples of effluent and wastewater were collected over a nine-week period (May–July 2017) (Table 1). In total, five samples of hospital effluent were collected from a 693-bed model 4 hospital, located in a large urban area in Ireland. The hospital provides an extensive range of services covering emergency, acute and outpatient care and treats on average 65,000 people annually. Samples of municipal wastewater pre (*n* = 5) and post (*n* = 4) entry of the hospital effluent to the main wastewater stream were also collected. All sample collection points are located within close proximity to one another and are illustrated in Fig. 1.

**Table 1:** Dates and sample types in which carbapenemase-producing *Enterobacterales* were detected.

Sample type	Sampling date	Species	Carbapenemase	MLST (Achtman)	Isolate ID
Pre-hospital wastewater	29th May 2017	*Escherichia coli*	NDM	ST617	NC28
Hospital effluent	15th May 2017	*Enterobacter cloacae complex*	OXA-48	Not sequenced	NC3
		*Enterobacter cloacae complex*	IMP	Not sequenced	NC7
		*Klebsiella pneumoniae*	OXA-48	ST3145	NC8
	29th May 2017	*Enterobacter cloacae complex*	VIM	Not sequenced	NC16
		*Citrobacter freundii*	KPC	Not sequenced	NC17
		*Enterobacter cloacae complex*	OXA-48	Not sequenced	NC20
		*Enterobacter cloacae complex*	IMP & OXA-48	Not sequenced	NC21A
		*Enterobacter cloacae complex*	IMP & OXA-48	Not sequenced	NC21B
		*Enterobacter cloacae complex*	OXA-48	Not sequenced	NC22
	12th June 2017	*Citrobacter freundii*	KPC	Not sequenced	NC46
		*Citrobacter freundii*	OXA-48	Not sequenced	NC49
		*Klebsiella pneumoniae*	IMP	ST3146	NC54
	26th June 2017	*Klebsiella oxytoca*	OXA-48	ST95	NC88
	11th July 2017	*Citrobacter freundii*	OXA-48	Not sequenced	NC116
Post-hospital wastewater	29th May 2017	*Klebsiella pneumoniae*	IMP	ST3146	NC37
		*Citrobacter freundii*	KPC	Not sequenced	NC38
	12th June 2017	*Klebsiella oxytoca*	VIM	ST202	NC66
		*Citrobacter freundii*	KPC	Not sequenced	NC67
		*Enterobacter cloacae complex*	OXA-48	Not sequenced	NC71
		*Klebsiella pneumoniae*	OXA-48	ST323	NC73
		*Citrobacter freundii*	OXA-48	Not sequenced	NC74
	11th July 2017	*Enterobacter cloacae complex*	OXA-48	Not sequenced	NC148

On each sampling date, one grab sample was collected (a minimum of 200 mL) from each of the sample collection points. Both pre-hospital and post-hospital wastewater samples were obtained by lowering a sterile 1 L glass bottle attached to string into a manhole at each of these collection points. Hospital effluent was obtained using the same technique and was collected from an onsite septic tank where the effluent is collected prior to its release into the main wastewater stream.

All samples were streaked onto Brilliance CRE Media (Oxoid) using a sterile cotton swab and incubated for 24 h at 37 °C. Following incubation all suspect CPE colonies were purified and identified using matrix-assisted laser desorption/ionization time of flight (MALDI-TOF) mass spectrometry in accordance with the manufacturer's instructions (Bruker; Biotyper version 4.1).

All *Enterobacterales* isolated were tested for susceptibility to 15 antimicrobial agents by disc diffusion in accordance with EUCAST criteria (EUCAST, 2018). These isolates were tested for susceptibility to the following antimicrobial agents; ciprofloxacin (5 μg), nalidixic acid (30 μg), ertapenem (10 μg), meropenem (10 μg), tetracycline (30 μg), trimpethoprim (5 μg), chloramphenicol (30 μg), gentamycin (10 μg), kanamycin (30 μg), streptomycin (10 μg), ampicillin (10 μg), ceftazidime (10 μg), cefoxitin (30 μg), cefotaxime (5 μg) and cefpodoxime (10 μg) (EUCAST, 2018). For quality control, *Klebsiella pneumoniae* strain ATCC 700603 and *Escherichia coli* strain ATCC 25922 were used.

All ertapenem and/or meropenem non-susceptible isolates were examined for carbapenemase-encoding genes using real-time PCR. Isolates were tested for the presence of *bla*_KPC_, *bla*_OXA-48_ and *bla*_NDM_ using PCR assays reported previously (CDC, 2011; Swayne et al., 2011) and for *bla*_VIM_ and *bla*_IMP_ using a duplex assay designed by the National Carbapenemase-producing *Enterobacterales* Reference Laboratory Service (NCPERLS) (Unpublished data; NCPERLS).

All carbapenemase-producing *E. coli* (*n* = 1), *K. pneumoniae* (*n* = 4) and *K. oxytoca* (*n* = 2) were sequenced (paired-end sequencing, read length 150 base pairs) using the Illumina HiSeq 500 platform. Genomes were hosted within the PubMLST BIGSdb database for *E. coli* and *K. oxytoca* (Jolley et al., 2018) and within the Pasteur *Klebsiella* BIGSdb database for *K. pneumoniae* (http://bigsdb.pasteur.fr/klebsiella), and made publicly available in the European Nucleotide Archive (https://www.ebi.ac.uk/ena). Genomic analyses were performed using the BIGSdb platform (Jolley et al., 2018), and the Centre for Genomic Epidemiology bacterial analyses pipeline (Thomsen et al., 2016).

## 3 Results

In total, 142 isolates of *Enterobacterales* were collected from hospital effluent (*n* = 65, 46%), pre-hospital (*n* = 31, 22%) and post-hospital wastewater (*n* = 46, 32%) samples. Antimicrobial susceptibility testing revealed 64/142 *Enterobacterales* (hospital effluent (*n* = 38, 59%), post-hospital wastewater (*n* = 17, 27%), pre-hospital wastewater (*n* = 9, 14%)) were non-susceptible to ertapenem and/or meropenem.

Of the 64 *Enterobacterales* that were non-susceptible to carbapenems, 36% (*n* = 23) were found to be carbapenemase-producers. CPE were detected in 100% (*n* = 5) of hospital effluent, 75% (n = 3) of post-hospital wastewater and 20% (n = 1) of pre-hospital wastewater samples. Table 1. provides an overview of the sample collection dates, sites and variants of carbapenemase detected. A total of 14/23 (61%) CPE (*K. pneumoniae* (n = 2), *K. oxytoca* (n = 1), *Citrobacter freundii* (n = 4) and *Enterobacter cloacae complex* (*n* = 7)) were collected from five hospital effluent samples. Of these 14 CPE, 12 harboured a single carbapenemase-encoding gene; *bla*_OXA-48_ (n = 7), *bla*_KPC_ (n = 2), *bla*_IMP_ (n = 2) and *bla*_VIM_ (*n* = 1), while the remaining two (both *E. cloacae complex*) harboured two carbapenemase-encoding genes; both *bla*_IMP_ and *bla*_OXA-48_. Eight CPE were isolated from post-hospital wastewater samples, all of which harboured a single carbapenemase-encoding gene; two *K. pneumoniae* (1 *bla*_OXA-48_, 1 *bla*_IMP_), one *K. oxytoca* (*bla*_VIM_), three *C. freundii* (2 *bla*_KPC_, 1 *bla*_OXA-48_), and two *E. cloacae complex* (both *bla*_OXA-48_). Only one CPE, an NDM-producing *E. coli,* was recovered from pre-hospital wastewater.

**Fig. 2:**
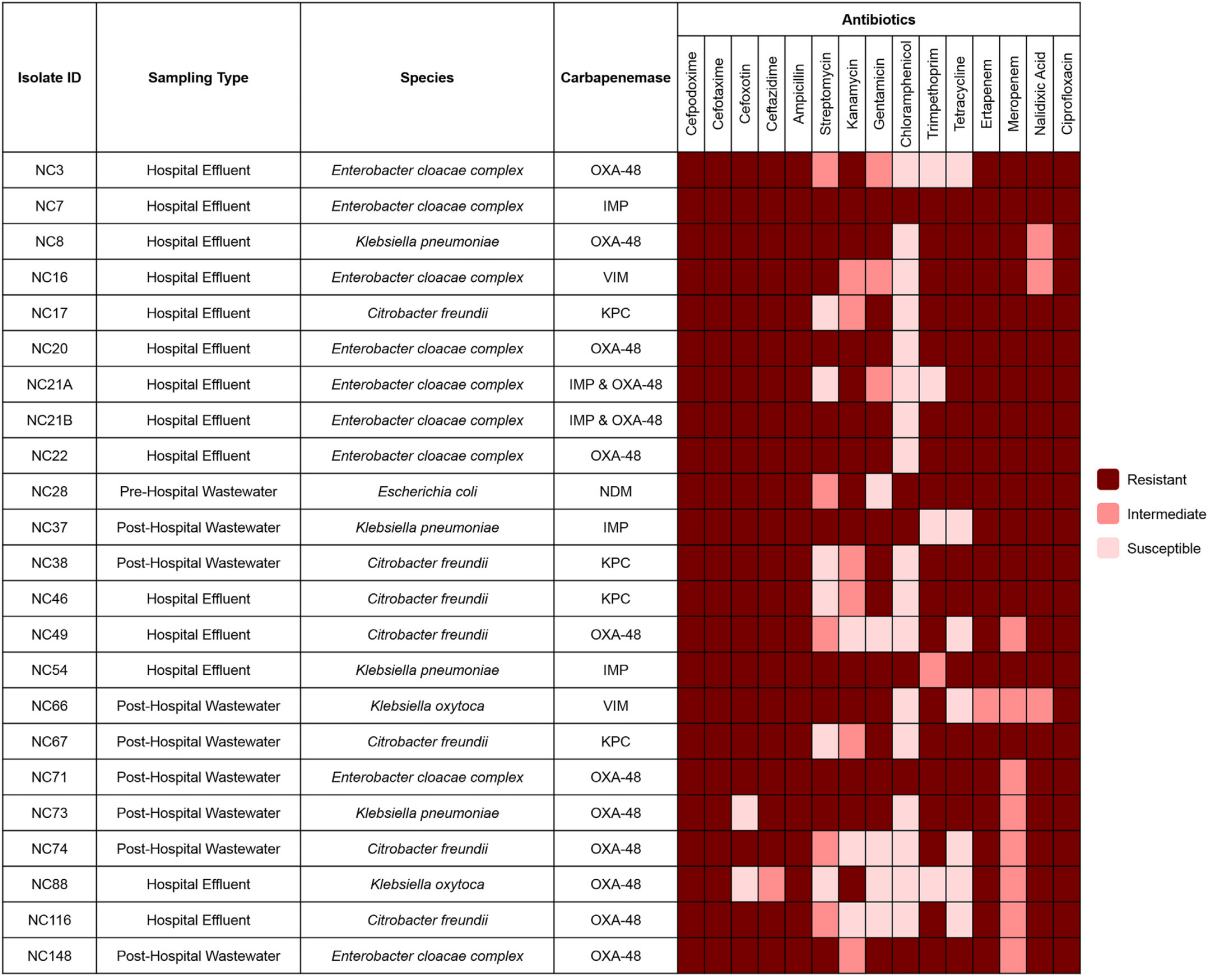
Antimicrobial susceptibility profiles of carbapenemase-producing *Enterobacterales* isolated.

Antimicrobial resistance profiles of CPE isolated from hospital effluent, pre-hospital and post-hospital wastewater samples are reported in Fig. 2. All CPE (*n* = 23) isolated from all three sources were multi-drug resistant (i.e. resistant to 3 or more antibiotic classes), with all isolates demonstrating non-susceptibility to a minimum of 9/15 antimicrobial agents tested. Four isolates demonstrated non-susceptibility to all 15 antimicrobial agents and included IMP-producing *E. cloacae* (*n* = 1) and IMP-producing *K. pneumoniae* (n = 1) from hospital effluent samples and OXA-48-producing *E. cloacae* (n = 2) from post-hospital samples. A high rate of non-susceptibility against all antimicrobials tested was noted, with the exception of chloramphenicol, with 17/23 CPE demonstrating susceptibility.

All carbapenemase-producing *E. coli* (n = 1), *K. pneumoniae* (*n* = 4) and *K. oxytoca* (n = 2) isolates detected were selected for whole genome sequence analysis (Table 1). In silico analyses revealed significant diversity, with all isolates but two having distinct STs (both IMP-producing *K. pneumoniae* isolated from hospital effluent and post-hospital wastewater on two different occasions were ST3146).

## 4 Discussion

Carbapenemase-producing *Enterobacterales* pose a huge threat to human health due to the limited treatment options available. In recent years a huge emphasis has been placed on the One Health concept and the importance of recognising the potential roles of animals and the environment in transmission, persistence and dissemination of ARB, including CPE.

Hospitals are a key focus for ARB emergence and dissemination. Large-scale antibiotic usage in the hospital setting has contributed to an increase in antibiotic residues and ARB in hospital wastewater systems (Morris et al., 2016; WHO, 2014; Kümmerer, 2003). Therefore, wastewater serves as a potential route for transfer of CPE from hospitals into the environment.

In Ireland, similar to many countries across Europe, hospital effluent is generally released untreated into the urban wastewater stream for treatment at an urban WWTP prior to discharge into the environment (Morris et al., 2016). The prime focus of urban wastewater treatment is to eliminate organic and inorganic contaminants; however, it is not designed to eliminate antibiotic residues or ARB (Morris et al., 2016). Antimicrobial resistant bacteria have been detected in treated effluent of urban WWTPs previously (Morris et al., 2016).

The World Health Organisation (WHO, 2014) recommend that hospitals have onsite facilities for the pre-treatment of hospital effluent prior to its release into the general wastewater stream in order to eliminate the presence of hazardous components including microbiological pathogens, radioactive drugs, toxic chemicals and antibiotics. In recent years, some countries including Germany and Denmark, have initiated treatment of hospital effluent onsite for the removal of all harmful contaminants prior to release into the general wastewater stream (Krarup et al., 2015; MICRODYN-NADIR, 2012). Unfortunately, due to high-cost and operational challenges associated with onsite treatment of hospital effluent, progress on this issue has been slow in many other countries (Pauwels and Verstraete, 2006).

Wastewater treatment varies from country to country with higher-income countries generally treating up to 70%, middle-income countries treating 28%–38%, and lower-income countries treating as little as 8% of wastewater generated prior to its release into the environment (United Nations, 2017). On average, up to 80% of wastewater generated across the world today is being discharged into the environment untreated (United Nations, 2017). Currently in Ireland, although in general wastewater is treated at an urban WWTP prior to discharge, there are 38 points across the country where untreated wastewater is still being emitted directly into the environment (EPA, 2018). Mahon et al. (2017) reported the first detection of CPE in seawater in Europe in 2017 and highlighted the discharge of untreated wastewater into the environment as the most likely source of CPE contamination. There is an associated public health risk with the discharge of inadequately treated or untreated wastewaters, especially those containing hospital effluent. This is due to the potential presence of harmful contaminants such as radioactive drugs, toxic chemicals, antibiotics and antimicrobial resistant bacteria.

This study found that CPE is more commonly detected in both hospital effluent and post-hospital wastewater than in pre-hospital wastewater. Four out of the five carbapenemases tested for; OXA-48, KPC, IMP and VIM were detected in hospital effluent and post-hospital wastewater samples. The most prevalent carbapenemase detected in both hospital effluent and post-hospital wastewater was OXA-48, which is the most commonly detected carbapenemase in clinical samples in Ireland at present (NCPERLS, 2018).

Other studies, which have assessed hospital effluent for the presence of CPE, have also reported similar findings. Zhang et al. (2012) reported KPC-producing *C. freundii* and *E. cloacae* in hospital effluent in China. In 2017, Zurfluh et al. (2017) reported the detection of a variety of CPE in the wastewater of a Swiss hospital, including OXA-48 and VIM-producing *E. coli*, OXA-48, VIM and NDM-producing *K. pneumoniae*, OXA-48 and VIM-producing *C. freundii,* OXA-48-producing *Citrobacter youngee,* OXA-48, NDM and VIM-producing *Citrobacter spp.,* OXA-48-producing *E. cloacae* and VIM-producing *Enterobacter aerogenes.* Gomi et al. (2018) also reported the isolation of CPE, including VIM and KPC-producing *Klebsiella spp*., NDM-producing *E. coli* and IMP and KPC-producing *E. cloacae*, in the wastewater of a Taiwanese hospital.

WGS analysis revealed a variety of MLST genotypes among the limited number of carbapenemase-producing *K. pneumoniae* isolates detected in both hospital and post-hospital wastewater samples; ST3145 (*n* = 1), ST3146 (*n* = 2) and ST323 (n = 1). To our knowledge, there have been no previous reports indicating the detection of carbapenemase-producing *K. pneumoniae* belonging to ST3145, ST3146 or ST323 in human or environmental samples. However, there have been reports of extended spectrum beta-lactamase (ESBL) producing *K. pneumoniae* ST323 detection in both human and environmental samples. The detection of the ESBL CTX-M-15 gene in *K. pneumoniae* isolates belonging to ST323 from specimens obtained from patients (i.e. urine, faecal, rectal), in both hospital and community settings has been reported previously, linking this particular sequence type with beta lactam resistance and highlighting its clinical significance (Dolejska et al., 2012; Gorrie et al., 2018; Mansour et al., 2015). Lepuschitz et al. (2019) reported the isolation of an ESBL-producing *Klebsiella pneumoniae* ST323 from an Austrian river. Following comparison of this isolate to an ESBL-producing *Klebsiella pneumoniae* ST323 isolated from a patient in an Austrian hospital, no genotypic match was found (Lepuschitz et al., 2019).

Analysis of the VIM-producing K. oxytoca (Isolate ID: NC66) detected in post-hospital wastewater revealed that it was ST202, which to our knowledge, has not been reportedly detected in clinical or environmental samples previously. The OXA-48-producing *K. oxytoca* (Isolate ID: NC88) isolated from hospital effluent was ST95. OXA-48-producing *K. oxytoca* belonging to ST95 has been previously detected in clinical specimens in the UK (Findlay et al., 2017). Other carbapenemase-producing *Enterobacterales* including VIM-producing *E. cloacae*, VIM-producing *C. freundii,* and NDM-producing *E. coli*, belonging to ST95 have also been detected in clinical specimens across the globe (Izdebski et al., 2018; Matsumura et al., 2017; Zhang et al., 2017), highlighting the clinical significance and dissemination of this sequence type.

Although NDM are one of the most commonly detected carbapenemases in clinical samples in Ireland at present (NCPERLS, 2018), in this study NDM-producing *Enterobacterales* was found to be present in wastewater on only one occasion. This was an NDM-5-producing *E. coli* belonging to ST617 and was detected in pre-hospital wastewater. The first NDM-5-producing *E. coli* was detected in a clinical isolate in the UK in 2011, and was ST648 (Hornsey et al., 2011). Zurfluh et al. (2017) reported the first finding of NDM-5-producing *E. coli* belonging to ST617, which was detected in municipal wastewater, as well as in the influent and effluent of a nearby wastewater treatment plant in Switzerland, in 2016 (Zurfluh et al., 2017). NDM-5-producing *E. coli* along with other NDM variants including NDM-1 and NDM-21, a newly reported variant derived from NDM-5, belonging to ST617, have been linked with the nosocomial setting (Gauthier et al., 2018; Liang et al., 2018; Liu et al., 2018; Torres-González et al., 2015; Zong et al., 2018). To date, the National CPE Reference Laboratory Service in Ireland has not identified clinical isolates of NDM-5 CPE.

Although in Ireland the number of infections caused by CPE is relatively low in comparison to other regions across the globe, it is increasing (Albiger et al., 2015; NCPERLS, 2017; NCPERLS, 2018). During the same time period, and in the same hospital in which sampling of hospital effluent took place, eight *bla*_OXA-48_, four *bla*_VIM_ and one *bla*_IMP_ were detected in clinical samples (Unpublished data; NCPERLS). Thus OXA-48, VIM and IMP-producing *Enterobacterales* were detected in both clinical samples and hospital effluent during this time. No KPC-producers were recovered in clinical samples; however, they were found in hospital effluent, potentially reflecting unidentified human carriage. We suggest that monitoring hospital effluent may merit further evaluation as an early signal of CPE dissemination in settings where CPE is perceived to be uncommon and patient screening is limited.

## 5 Conclusion

Hospital effluent is a significant source of CPE entering the environment. Monitoring of hospital effluent may have a value in signalling unrecognised colonisation in a hospital setting.

## Competing financial interests

The authors declare they have no actual or potential competing financial interests.
